# Policy integration fluctuations in China’s rural toilet revolution: typology and strategies for public health governance

**DOI:** 10.3389/fpubh.2026.1752521

**Published:** 2026-04-10

**Authors:** Minghua Jiang

**Affiliations:** School of Management, Guangzhou College of Commerce, Guangzhou, Guangdong, China

**Keywords:** China’s public health governance, policy integration fluctuations, policy integration strategies, toilet revolution, types of policy integration

## Abstract

China’s Rural Toilet Revolution, despite strong central support, reveals persistent challenges in sustaining policy integration within a complex, multi-level governance system. This study, based on process-tracing and fieldwork in Shaoguan, emphasizes the strategic agency of local governments in managing integration and develops a typology of integration fluctuations along two dimensions: vertical cross-level versus horizontal cross-departmental integration, and political authority versus social network drivers. Four patterns emerge—Bureaucratic Pressure-Driven, Differential Penetration-Driven, Institutional Regulation-Driven, and Network-Embedded Integration—demonstrating that fluctuations are not policy failures but adaptive responses to institutional tensions. Local governments actively reshape policy frames to align objectives, coordinate subsystems, and mitigate accountability risks. These frames serve a dual function: as tools for consensus-building and as sources of institutional legitimacy that shield actors from blame in cross-jurisdictional collaboration. The study advances policy theory by revealing how subnational actors navigate governance complexity and offers practical insights for designing resilient policy architectures in hierarchical systems where integration must be continuously renegotiated.

## 1 Introduction

Since 2003, the Chinese government has implemented a nationwide public health improvement strategy with a focus on rural areas, the success of which has hinged critically on achieving cross-cutting policy integration^1^. However, this process has encountered significant challenges in practice. On one hand, the scarcity of governance resources and the multiplicity of tasks in routine administration have compelled local governments to adopt strategies such as flexible implementation (1, 2) and collusive behavior (3), leading to fragmented policy execution that undermines the very foundation of integration. On the other hand, to address ineffective implementation at the grassroots level, higher-level governments have reinforced the pressure-driven system (known in Chinese as *Yalixing Tizhi*) through ceiling-level target management and high-stakes performance appraisals (4, 5). While this system enhances enforcement rigidity, it has also exacerbated the tension between multiple policy mandates and limited resources, rendering policy integration efforts highly uncertain—oscillating between symbolic integration to meet evaluation requirements (6) and selective integration in response to practical constraints (7), ultimately resulting in pronounced fluctuations in the policy integration process.

Such fluctuations, however, do not necessarily indicate a failure of policy integration or the policy itself. Rather, recurrent governance patterns are often manifestations of deep institutional tensions, rather than mere surface-level implementation problems. Existing literature has extensively documented the fragmented authoritarianism and implementation gaps in China, yet the dynamic mechanisms driving these fluctuations—specifically how local actors navigate the tension between political mandates and practical constraints—remain insufficiently explored. Unpacking the persistent fluctuations in China’s public health governance is therefore essential, as it not only advances theoretical understanding of the Chinese policy process but also offers practical pathways for enhancing governance effectiveness. Importantly, policy integration fluctuation should not be conflated with episodic deviations such as selective implementation or symbolic compliance. Rather, it denotes a patterned strategic recalibration—a recurrent reconfiguration of policy frames, actor roles, and instruments by local governments in response to persistent institutional tensions between central mandates and local capacities. This variation arises from three interrelated contextual factors: vertical resource asymmetry acts as a structural constraint, performance pressure from above serves as a key trigger, and dense horizontal relational networks enable adaptive coordination.

To address this gap, this study examines the implementation of China’s Rural Toilet Revolution—a nationwide public health campaign launched in 2018 to upgrade sanitation infrastructure in rural areas—as a critical case of policy integration under hierarchical governance and resource constraints. Drawing on fieldwork in Shaoguan Municipality, the analysis investigates how local governments navigate cross-departmental coordination challenges amid competing mandates and limited capacity. The central research question is: How do local actors dynamically adjust policy integration strategies in response to shifting institutional pressures, and what patterns can be identified in these adaptive responses? The study argues that observed fluctuations in integration efforts are not signs of failure but reflect context-sensitive governance adaptations. Accordingly, the paper contributes a typology of four integration modes and reframes fluctuation as a mechanism of adaptive governance.

The remainder of this paper is organized as follows. Section 2 reviews the literature on policy integration and adaptive governance, developing our analytical framework. Section 3 outlines the methodology and case selection. Sections 4 and 5 present the empirical findings and discuss their theoretical implications. The final section concludes with limitations and reflections on generalizability.

## 2 Policy integration and fluctuation in policy integration

### 2.1 From static integration to dynamic fluctuation

Policy integration has been widely conceptualized as the alignment of policy elements across multiple dimensions. Drawing on Candel and Biesbroek (8), scholars typically examine integration through four interrelated lenses: (1) coherence in policy frames (shared problem definitions and narratives), (2) inclusion of relevant actors within the subsystem, (3) consistency in policy goals, and (4) compatibility of policy instruments. This framework has proven valuable for diagnosing fragmentation and evaluating coordination efforts in complex policy arenas such as climate change, food security, and rural development (9, 10).

However, much of this literature implicitly assumes integration as a directional or even teleological process—moving from fragmentation toward greater coherence. In doing so, it often underemphasizes the temporal instability and strategic recalibration that characterize real-world implementation, particularly in contexts marked by hierarchical authority, resource scarcity, and institutional fragmentation. Empirical studies increasingly reveal that integration is not a stable state but a contested, reversible, and often cyclical endeavor (11, 12).

To account for this dynamism, recent scholarship has turned to mechanism-based analysis, which shifts focus from static outcomes to the causal processes through which actors navigate institutional tensions (13). Within this tradition, fluctuation in policy integration is not merely noise or failure, but may reflect deliberate adaptations to structural constraints. This aligns with perspectives on adaptive governance, which view iterative adjustment—not equilibrium—as the hallmark of effective action in complex systems (14, 15).

In the Chinese context, however, adaptation occurs not through open deliberation or consensus-building, but through strategic maneuvering within a tightly bounded political space (16, 17). Local actors therefore engage in what some scholars describe as policy integration fluctuation—a patterned oscillation between coordination modes that balances compliance with central mandates and responsiveness to local capacities, without overt defiance. This phenomenon calls for analytical approaches capable of tracing how specific strategies activate distinct integration mechanisms over time.

This study shifts analytical focus toward the dynamic unfolding of policy integration fluctuations. Drawing on a process-tracing analysis of the rural Toilet Revolution in Shaoguan City, Guangdong Province, this article seeks to (1) identify and classify distinct types of policy integration fluctuations and (2) uncover the strategic responses of local governments—as pivotal actors—in navigating diverse governance dilemmas. Through this analysis, the article elucidates the underlying process mechanisms of policy integration fluctuations and underscores the central role of actors within them, thus contributing to a more dynamic and comprehensive theoretical framework for understanding the complexity of China’s public policy processes.

### 2.2 A typological framework for analyzing fluctuation dynamics

This article examines the fluctuating nature of policy integration—not as random variation, but as patterned shifts that unfold across the policy process. Understanding these dynamics requires a systematic typology of their underlying forms. To this end, the study develops a two-dimensional framework that reinterprets Candel and Biesbroek (8) multi-dimensional approach in light of China’s distinctive governance context.

The first dimension is integration domain, which distinguishes whether coordination occurs primarily across vertical levels or among horizontal departments. China’s policy implementation operates within a unitary system structured around the *tiao-kuai* (vertical-horizontal) duality. The “*tiao*” refers to vertically integrated functional departments that ensure sectoral consistency from central to local levels, while the “*kuai*” denotes territorial governments responsible for comprehensive jurisdictional governance and cross-sectoral coordination within their boundaries (18). This institutional arrangement generates a persistent tension between centralized control and local adaptability, shaping two analytically distinct domains of integration. Vertical cross-level integration, concerning the transmission and adaptation of central mandates down administrative tiers; and Horizontal cross-departmental integration, involving coordination among co-located functional agencies under local government leadership.

The second dimension concerns integration drivers—the mechanisms that initiate and sustain alignment across levels and sectors. Drawing on Cejudo and Trein (19), two ideal-typical drivers are identified: formal political authority and informal social networks. Authority-driven integration relies on top-down instruments such as performance evaluations, administrative orders, and fiscal control, enforcing compliance through hierarchical accountability. In contrast, network-driven integration emerges from relational ties—trust, shared identity, or long-term collaboration—enabling flexible, context-sensitive coordination that adapts to local complexities.

These two dimensions are analytically indispensable for capturing policy integration fluctuation in China’s governance context. Conventional integration theory focuses on outcomes—coherence, consistency, inclusion—but offers limited tools to explain why and how integration waxes and wanes over time. Fluctuation, by contrast, is fundamentally a relational and structural phenomenon: it arises from the interplay between (1) where coordination is demanded (the institutional arena of tension) and (2) how alignment is achieved (the available mechanisms of action). In China, the tiao-kuai duality defines the primary fault line of policy implementation: vertical functional departments (*tiao*) pull toward sectoral uniformity, while territorial governments (*kuai*) push for localized synthesis. This makes integration domain—vertical versus horizontal—the key axis along which institutional conflict manifests. Simultaneously, Chinese governance operates through a dual logic: formal authority ensures political control, while informal networks enable practical adaptation under ambiguity. Thus, integration drivers—authority versus networks—determine the repertoire of strategies actors can deploy. It is precisely at the intersection of these two structurally grounded axes that patterned fluctuations emerge, as local actors navigate competing imperatives without exiting the system. Alternative dimensions (e.g., policy sectors or temporal phases) may describe variation, but only this pairing captures the causal architecture of fluctuation in hierarchical, fragmented settings. Combining these two dimensions yields a 2 × 2 typology (see Table 1).

**Table 1:** Typological Framework of Rural Public Policy Integration in China.

		**Integration Drivers**	
		Political Authority-Driven	Social Network-Driven
**Integration Domains**	Vertical Cross-Level	Bureaucratic Pressure-Driven Integration (1) Policy implementation is enforced through top-down administrative mandates; (2) Driven by hierarchical political authority and performance accountability; (3) Embodies a command-obedience model of integration.	Differential Penetration-Driven Integration (1) Policy objectives spread gradually from the center to the periphery through informal social networks; (2) Driven by trust, identity, and personal ties within social networks; (3) Integration intensity varies with relational proximity, exhibiting a concentric pattern of diminishing penetration.
	Horizontal Cross-Departmental	Institutional Regulation-Driven Integration (1) Cross-departmental coordination is achieved through formal inter-agency mechanisms; (2) Driven by institutional mandates and compliance pressures from higher-level authorities; (3) Aims to overcome bureaucratic silos and achieve policy synergy.	Network-Embedded Integration (1) Spontaneous collaboration emerges through informal ties based on shared interests or long-term cooperation; (2) Driven by mutual trust, reciprocity, and relational capital; (3) Characterized by flexibility, adaptability, and context-sensitive coordination.

This framework is not merely descriptive; it serves as an analytical lens to trace how local governments strategically shift between modes in response to institutional pressures. While the typology structures the analysis, the study retains Candel and Biesbroek (8) four-dimensional diagnostic criteria—policy frames, subsystem involvement, goals, and instruments—to assess how each strategy reconfigures integration outcomes.

To uncover these dynamics, the study employs process tracing in an in-depth case of the Rural Toilet Revolution in Shaoguan City. Fieldwork began in July 2022, with 56 semi-structured interviews conducted across two rounds (2022–2023) with officials, village cadres, residents, and contractors, supplemented by policy documents and meeting records. Triangulation enhances reliability, while attention to timing, actor accounts, and alternative explanations strengthens causal inference. Although findings are context-specific, they reveal plausible micro-mechanisms of fluctuation under conditions of resource scarcity and institutional fragmentation—challenges widespread in rural governance.

## 3 Toward policy integration: the evolution of rural public health governance in China

**Figure 1:**
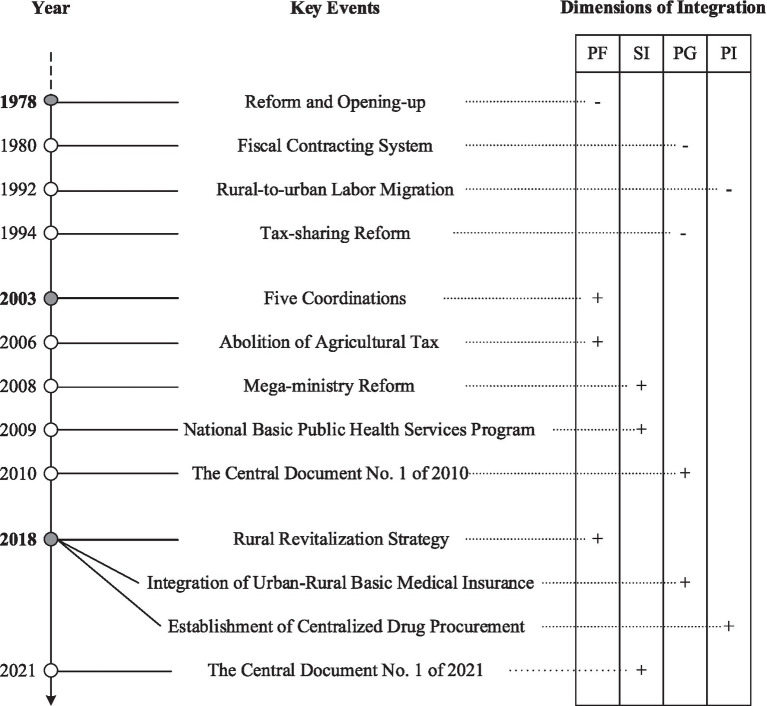
Schematic diagram of the evolution of rural public health governance in China. Solid dots represent key events that signify phase divisions, while hollow dots indicate other key events. According to Candel and Biesbroek (8)‘s discussion on dimensions of policy integration, PF, SI, PG, and PI stand for policy framework, subsystem involvement, policy goals, and policy instruments, respectively. “+” indicates that the event promoted the integration of rural public health policies, while “−” indicates the opposite.

Public policy, as a response to evolving societal demands, reflects the defining features of its historical context. From the founding of the People’s Republic of China to the pre-reform era, rural public health policy was characterized by pronounced politicization, frequently subordinated to overarching ideological campaigns rather than systematic public health objectives. The analysis is anchored in the pivotal moment of 1978—the onset of China’s reform and opening-up—as the historical starting point for the transformation of rural public health governance. Drawing on key policy milestones, this paper delineates the evolution into three distinct stages (see Figure 1). By analyzing the core characteristics of each stage through the lens of policy integration, this section establishes a macro-level framework that situates and informs the detailed case studies that follow.

### 3.1 1978–2002: governance fragmentation under market rationalism

This phase commenced with the 1978 reform and opening-up. While promoting political decentralization, market rationalism gave rise to increasing misalignment between central and local governments over rural public health governance objectives. The implementation of the fiscal contracting system (*Caizheng Baogangzhi*)^2^ and subsequent tax-sharing reform (*Fenshuizhi*)^3^ tightly linked local government revenues to economic performance, creating powerful incentives to prioritize GDP growth (20). As a result, local governments were structurally incentivized to prioritize investments with immediate economic returns—such as attracting foreign and domestic investment and advancing industrial development—at the expense of rural public health, a long-term, low-visibility domain with delayed benefits (21). Although the central government consistently emphasized social equity and the equalization of basic public services, central directives were frequently subject to selective or symbolic implementation at the local level. This pattern solidified a pronounced center-local misalignment in public health governance, laying the structural foundations for fragmented and under-resourced rural health systems.

Beyond intergovernmental tensions, sectoral fragmentation further weakened governance coherence. During this period, rural public health governance had not emerged as a cross-sectoral policy priority. Policy formulation remained predominantly confined to the Ministry of Health, with other functional departments lacking systematic integration of public health considerations into their decision-making (22). Cross-sectoral coordination was *ad hoc* and incidental, perpetuating entrenched sectoral fragmentation. Meanwhile, the industrial and commercial sectors—despite their potential to function as complementary governance actors—remained marginal in practice. While township enterprises flourished and had become economic pillars in certain rural areas, they were oriented toward profit maximization rather than public service provision (23). Even more concerning, some local governments relaxed environmental regulations to attract investment, thereby undermining rural ecological conditions and population health, and further entrenching governance fragmentation and inefficiency (24).

Compounding these institutional challenges, the scarcity of effective policy instruments constrained systemic responses. The implementation of the Household Responsibility System (*Jiating Lianchanchengbao Zerenzhi*) released vast rural labor forces into non-agricultural sectors, culminating in a massive wave of rural-to-urban migration by 1992. In a context of limited governance resources, this demographic shift further eroded the collective economic base of villages, undermining the financial sustainability of village health clinics and accelerating the decay of the primary healthcare network. In response, while local and higher-level governments attempted to arrest this decline through administrative directives and modest fiscal transfers, they lacked a robust repertoire of modern policy instruments—particularly market-based incentives and mechanisms for social mobilization (25). This gap limited their capacity to mobilize societal resources and establish inclusive, multi-stakeholder governance arrangements, leaving rural public health services severely compromised in both coverage and quality. This fragmentation created enduring center-local misalignment, setting the stage for later fluctuations when new integration mandates clashed with entrenched local incentives.

### 3.2 2003–2017: the rise of the joined-up government approach

The second phase was catalyzed by the introduction of the Five Coordinations (*Wuge Tongchou*)^4^ national strategy in 2003—a pivotal shift in China’s developmental trajectory. This strategic reorientation emerged in response to a confluence of socioeconomic crises: the Asian financial crisis, plummeting agricultural prices, and heavy fiscal burdens on rural households precipitated a sustained decline in rural incomes from 1998 to 2000 (26). Although the central government initiated tax reforms and limited compensatory measures from 2001 onward, these interventions failed to address the underlying structural crisis in the countryside. In turn, the central government elevated “coordinating urban-rural development” to the apex of the national policy agenda, signaling a fundamental transformation in the state-rural relationship—from one of resource extraction to one of state-led provision (27). The complete abolition of the agricultural tax in 2006 emerged as the defining symbol of this paradigm shift.

To address entrenched governance fragmentation, the central government advanced rural public health governance toward a joined-up government model by strengthening both decision-making coordination and implementation coordination (28). This approach sought to dismantle bureaucratic silos and integrate governance resources across jurisdictional boundaries, thereby enhancing policy coherence and service delivery effectiveness. Decision-making coordination was elevated to a political imperative, requiring mutual consultation among central ministries and between central and local governments during policy formulation. Central directives mandated vertical and horizontal alignment of objectives to ensure policy consistency across sectors (29). A key example was the 2010 directive on urban–rural coordinated development, which specified concrete tasks to harmonize policy goals—including those in rural public health—across departments and administrative levels (30).

At the implementation level, vertical coordination was strengthened through the 2008 mega-ministry reform (*Dabuzhi Gaige*), which not only streamlined the administrative structure of the Ministry of Agriculture from a three-tier hierarchy (ministry-directorate-division) to a two-tier framework (mega-ministry-directorate), but also consolidated previously fragmented agricultural-related functions into a single institutional entity, significantly enhancing the efficiency of policy transmission in rural sectors (31). In parallel, the 2009 launch of the National Basic Public Health Services Program (NBPSP) exemplified horizontal coordination: by integrating previously fragmented functions across agencies, it established a formal, inter-ministerial collaboration mechanism involving multiple central government departments. This initiative enabled, for the first time, the nationwide provision of standardized primary public health services with urban–rural parity (32). Through this dual coordination in both decision-making and implementation, rural public health governance in China underwent an initial institutional transformation—from fragmentation toward integration—and laid the foundational architecture of a joined-up government framework. While joined-up mechanisms improved coordination, their reliance on top-down pressure planted seeds for future oscillation between compliance and adaptation.

### 3.3 2018–present: deepening and expansion of the joined-up government paradigm

The third phase, beginning in 2018, is defined by the nationwide implementation of the Rural Revitalization Strategy.^5^ As a national top-level design, this initiative not only extends the collaborative governance framework (14) but also introduces significant advances in governance philosophy and institutional architecture. By demanding greater goal alignment and actional coordination, it elevates joined-up government from an operational approach to a normalized governance paradigm, thereby laying a solid foundation for holistic governance.

This transformation was driven primarily by intense political mobilization. To consolidate poverty alleviation achievements, the central government elevated rural revitalization to a strategic national priority. Through repeated high-level endorsements and ideological framing, the strategy was transformed into a hegemonic discourse—a dominant narrative that aligns institutional behavior and mobilizes societal compliance (33). Under the impetus of this discourse, China’s rural public health governance underwent substantial reconfiguration across both horizontal and vertical dimensions.

In the mid-2010s, coordination mechanisms and policy innovation within the state apparatus reached a new level of institutionalization. Horizontally, the integration of urban and rural basic medical insurance—initiated in 2016 and largely completed by 2018—ended long-standing divides between the New Rural Cooperative Medical Scheme (launched in 2003) and urban insurance systems. Standardized frameworks for financing, reimbursement, and essential drugs shifted coverage from fragmentation toward coherence and equity (34). A key turning point was the 2018 consolidation of public health functions across agencies into a single authority. A key turning point was the central government’s decision in 2018 to establish a dedicated institution responsible for public health decision-making. This reorganization ended decades of functional dispersion and enabled a whole-of-health governance model, aligning previously divergent sectoral objectives and improving policy coherence. From this foundation, new instruments emerged to address systemic inefficiencies. Notably, the centralized bulk procurement system for pharmaceuticals—introduced in 2018—uses state purchasing power to reduce drug prices (35). By embedding market mechanisms within coordinated governance, it has significantly lowered out-of-pocket costs, especially for rural households, demonstrating how institutional integration can advance both equity and efficiency in service delivery.

Yet the most transformative shift in holistic governance lies beyond the state: in the transcendence of the government-society divide and the diversification of governance actors. While the Rural Revitalization Strategy channeled vast resources into rural areas through pro-agricultural programs, grassroots governments could no longer manage rural populations through administrative directives alone (36). In response, and to better align with the strategic goals of rural revitalization, the central government reoriented its rural governance paradigm in 2021, strategically promoting villagers’ self-governance organizations as pivotal vehicles for resource delivery, demand articulation, and consultative co-governance. In this context, community-based bodies—such as village councils and deliberative assemblies—have proliferated. These organizations not only assume substantive responsibilities but have also become indispensable in the provision of public health services (37). Their deep engagement reflects a transformative phase in rural governance—the deepening of inter-agency and inter-level coordination and the expansion of the joined-up government paradigm beyond state boundaries to incorporate self-governance organizations as co-producing actors, thereby institutionalizing a more integrated and participatory model of public health delivery. The holistic paradigm intensified expectations for integration, yet its ambitious scope—coupled with persistent resource gaps—generated new tensions that local governments would navigate through fluctuating strategies.

The evolution of rural public health governance in China reveals a clear trajectory: from fragmentation under market rationalism, through the reassertion of state coordination in the joined-up government era, to an emerging model of holistic governance under the Rural Revitalization Strategy. This transformation was driven by the structural failures of decentralized, sectorally siloed governance in the 1980s and 1990s—where fiscal incentives and administrative fragmentation undermined rural health provision. In response, the state progressively reasserted its integrative capacity, first through top-down coordination mechanisms in the 2000s and 2010s, and later through institutional consolidation and cross-sectoral integration after 2018. Central to this evolution has been the deepening of policy integration—across levels of government, functional domains, and state-society boundaries. The shift from fragmented service delivery to coordinated, and ultimately co-produced, governance reflects not only a technical refinement of policy instruments but also a broader transformation in the state’s governing logic: from extraction to investment, from control to collaboration, and from bureaucracy to integration. This trajectory underscores the Chinese state’s adaptive capacity in recalibrating governance boundaries to meet complex societal challenges. The following section examines the Toilet Revolution in Shaoguan as a case study, analyzing how this macro-level shift manifests in local practice, with a focus on patterns of policy integration and the strategic roles of key actors.

## 4 The implementation process of the toilet revolution policy in Shaoguan

To promote rural development, China’s central government launched the Rural Toilet Revolution^6^ in 2018. Shaoguan Municipality responded in early 2019 with an action plan that set ambitious targets but provided insufficient funding and personnel, creating chronic resource deficits. This scarcity triggered strategic contestation among actors across levels, generating dynamic fluctuations in policy integration—a process shaped less by top-down control than by adaptive bargaining under constraint.

The Shaoguan Agriculture and Rural Bureau (ARB) was formally designated as the lead agency, tasked with standard-setting, resource allocation, and project oversight. It was further granted exclusive authority to operate a cross-departmental data platform, positioning it as the sole channel for reporting implementation progress to municipal leadership. In this context, the ARB emerged as the dominant involved subsystem in policy integration. Yet this dominance was neither automatic nor uncontested. It stemmed less from formal mandate than from the bureau’s technical expertise, deep embeddedness in rural governance, and strategic control over performance information. At the same time, its authority faced persistent pushback: functional departments resisted coordination on jurisdictional grounds, while county governments viewed ARB oversight as an encroachment on local discretion—particularly when assigned targets far exceeded available resources. Thus, ARB’s leadership was not an institutional given, but a contingent outcome of continuous negotiation amid competing governance logics.

### 4.1 Bureaucratic pressure-driven integration: administrative authority and vertical goal alignment

In implementing the Rural Toilet Revolution, Shaoguan Municipality confronted a fundamental governance challenge: the vertical misalignment of policy objectives, rooted in systemic resource constraints. As a complex rural public policy initiative, the program encountered two interrelated tensions across administrative hierarchies. First, higher-level funding mandates far exceeded the fiscal capacity of several county governments, creating significant implementation barriers. Second, standardized technical specifications and long-term maintenance requirements clashed with the operational autonomy that local administrations need to adapt to heterogeneous conditions. These constraints led local officials to engage in selective implementation or informal adaptations—strategies that diluted policy coherence during vertical transmission and eroded overall efficacy. To counteract this fragmentation, the ARB engineered a bureaucratic pressure-driven integration mechanism, leveraging political authority to enforce top-down policy alignment.

Central to these strategies was the institutionalization of target responsibility agreements. Initiated by the ARB and formalized through municipal decree, binding performance contracts were signed with county and district governments, translating broad policy directives into specific, measurable, and time-bound administrative tasks. These agreements set quantitative targets—such as toilet construction quotas—and defined qualitative benchmarks in construction standards and completion timelines. By codifying responsibilities and performance metrics, the contracts established a formal policy frame, converting strategic goals into enforceable obligations and establishing the institutional foundation for vertical policy coherence.

Complementing the target-responsibility system, the ARB introduced a designated contact system that linked senior municipal officials directly to local implementation. Under this arrangement, the mayor and other top leaders assumed personal oversight of designated counties or districts, with assignments tailored to implementation complexity and regional needs. This mechanism embedded high-level political authority within frontline governance, transforming abstract policy commitments into traceable individual accountability. While the target agreements provided a rule-based framework, the contact system introduced sustained political pressure and active supervision, strengthening the chain of command from policy design to execution.

Beyond formal mechanisms, the ARB also institutionalized ritualized practices to reinforce policy legitimacy and internalize goal alignment. The signing of responsibility agreements was elevated into a ceremonial event, broadcast live to amplify public scrutiny and signal political resolve. Concurrently, officials were organized into study tours to model sites in high-performing cities, fostering a culture of emulation and competitive learning. These performative acts functioned not merely as symbolic gestures, but as tools of cognitive and emotional alignment, encouraging local cadres to identify with the policy goals.

Collectively, this model exemplifies the deep penetration of political authority into routine administration. Through the interplay of contractual obligations, leadership engagement, and symbolic rituals, the ARB succeeded in transforming top-down mandates into binding constraints on local governments, achieving a high degree of formal policy goal coherence. Yet this integration strategy did not resolve the underlying tensions of implementation. Constrained by limited fiscal resources, many counties restricted reforms to pilot zones after signing agreements—engaging in symbolic compliance that fell far short of universal coverage. Moreover, to meet targets efficiently and minimize risk, local authorities routinely replicated provincial technical standards without modification, disregarding local ecological and social conditions. In high-altitude mountainous regions, for instance, standardized toilet facilities froze during winter, requiring costly reconstruction and generating public dissatisfaction. These outcomes reveal a critical governance trade-off: while bureaucratic pressure ensures short-term compliance, it risks undermining local adaptive capacity and long-term policy sustainability. The very mechanisms that secure vertical alignment may simultaneously erode the flexibility needed for effective local governance. Yet the rigidity of bureaucratic pressure generated local resistance and maladaptation, prompting the ARB to seek more flexible horizontal coordination mechanisms.

### 4.2 Institutional regulation-driven integration: the reconfiguration of horizontal relations

In implementing the Rural Toilet Revolution, Shaoguan Municipality faced not only vertical implementation tensions but also profound challenges in horizontal interagency coordination. Within China’s *Tiao-Kuai* administrative structure, functional specialization inherently produces rigid jurisdictional boundaries—giving rise to policy silos marked by conflicting technical standards, redundant inspection procedures, and, most critically, fragmented fund allocation. To overcome these structural barriers, the ARB, as the dominant subsystem, initiated institutional regulation-driven integration strategies to reconfigure interdepartmental authority and responsibility relations, laying the foundation for more cohesive multi-agency policy frame.

To operationalize horizontal integration, the ARB undertook a formal decomposition of policy goals and redefined interdepartmental roles to address resource-task imbalances. Recognizing the impracticality of centralized execution, it shifted from unilateral control to functional differentiation, redistributing implementation responsibilities according to sectoral expertise. While retaining oversight of rural household toilets, the ARB delegated public facility construction to specialized agencies: township public toilets to the Housing and Urban–Rural Development Bureau (HUB), school toilets to the Education Bureau (EDB), and tourist-area toilets to the Culture, Radio, Television, Tourism and Sports Bureau (CTB). This reallocation relieved the ARB’s operational burdens and enhanced task-agency fit by aligning responsibilities with functional mandates. Through this arrangement, the single-department policy agenda was transformed into a structured, multi-agency policy frame, codified in municipal supplementary directives.

The ARB established a formal joint conference system as the primary coordination mechanism to sustain interdepartmental involvement. More than a forum for information exchange, this platform functioned as a strategic agenda-setting institution, capable of depoliticizing conflicts by reframing them as technical issues open to negotiated resolution. When the HUB questioned the budget’s feasibility—citing volatile construction material prices—the underlying concern was resistance to the ARB’s centralized procurement authority. Through the joint conference, this potentially disruptive dispute was redirected toward technical adjustments in procurement protocols and cost benchmarks. Over time, such institutionalized deliberation cultivated incremental trust and mutual accommodation, reinforcing a collaborative ethos essential to the framework’s long-term viability.

Institutional regulation-driven integration transcends conventional administrative coordination. It constitutes a deliberate restructuring of horizontal governance relations through formal rules, aiming to construct and sustain a polycentric policy network. Yet this model entails an inherent governance tension: its effectiveness hinges on the dominant subsystem’s ability to balance authoritative direction with inclusive participation. If the ARB’s authority is too weak, collaboration risks devolving into symbolic participation—ritualistic meetings without substantive coordination. Conversely, excessive control may stifle initiative and innovation across departments, fostering dependency and resentment. Such imbalances can erode interagency trust, dampen cooperation, and ultimately undermine the resilience of the policy frame. Therefore, the success of this integrational model depends not solely on institutional design, but on the dynamic calibration of authority and autonomy. However, formal inter-agency regulation proved insufficient to scale grassroots innovations, leading the ARB to leverage social networks for broader mobilization.

### 4.3 Differential penetration-driven integration: social relations and cross-level integration

When policy resources are scarce and implementation flexibility is constrained, even within a pressure-driven system, frontline implementers may develop low performance expectations, eroding their identification to with policy objectives. This psychological detachment often manifests as disengagement or passive resistance, thereby undermining the prospects for sustained policy integration. In the early stages of implementation, shortly after the initial mobilization, Shaoguan’s Rural Toilet Revolution encountered precisely this challenge. Rather than reinforcing top-down control, the ARB pursued a dual-track strategy: first, allowing informal practices to coalesce into a new policy framework; second, designing new policy instruments grounded in this framework, thereby expanding the flexible institutional space available for local implementation.

At the core of this approach was the strategic mobilization of social capital through informal mechanisms under conditions of fiscal scarcity and performance pressure. In Xinshao Town—a township under Zhenjiang District—local officials explored two innovative pathways for private sector engagement. First, in urban areas, they authorized social capital to operate and maintain public toilets through regulated revenue-generation models. Second, in rural areas, they adopted a “build-first, subsidize-later” model: enterprises advanced funds for the construction or renovation of household toilets, with government subsidies disbursed only after successful inspection and acceptance. As a regulatory body, the ARB refrained from imposing rigid compliance checks; instead, it collaborated with the Zhenjiang Government and other municipal departments to designate these initiatives as experimental pilots, thereby creating regulatory flexibility for local adaptation.

Crucially, high-level political endorsement transformed these local experiments into a policy frame. To reconcile grassroots innovation with rigid higher-level mandates, the ARB and Zhenjiang Government jointly facilitated a site visit by the Governor of Guangdong Province to Xinshao. Recognizing the model’s effectiveness in aligning incentives among government, enterprises, and rural households—while simultaneously alleviating financial pressure on local governments and reducing household expenditures—the Governor publicly endorsed it as a scalable innovation in rural governance. This endorsement functioned as a mechanism of institutionalization-by-approval, converting an informal, localized practice into a legitimate policy frame.

This new policy frame laid the foundation for the introduction of substantive policy instruments. Following the inspection, the ARB issued formal implementation guidelines for social participation in the Rural Toilet Revolution. These guidelines specified the scope of market involvement in public toilet operations, established eligibility criteria for private investors, and standardized subsidy benchmarks for rural households. By codifying previously *ad hoc* arrangements, the guidelines not only provided local governments with standardized operating procedures—clarifying pathways for social capital engagement and associated incentives—but also systematically corrected implementation deviations and resource misallocation observed during the pilot phase.

The essence of differential penetration-driven integration lies in the strategic deployment of informal mechanisms to create flexibility within rigid bureaucratic structures. It leverages political authority as an implicit guarantee, enabling a transition from rigid control to resilient adaptation. Yet this model entails significant risks. The semi-institutionalization process—driven by high-level inspection and endorsement—may generate new path dependencies. Overreliance on political validation risks transforming grassroots innovation into performative governance, where local initiatives are designed not for local needs, but for the expectations of visiting officials. While differential penetration enabled local experimentation, its dependence on political endorsement limited institutionalization—necessitating a city-wide network to sustain momentum.

### 4.4 Network-embedded integration: social capital driven inter-agency collaboration

While the Xinshao case demonstrated how local governments could leverage social capital to advance the Toilet Revolution, its practices remained localized experiments led by the Zhenjiang government and the ARB. Despite high-level political support, these initiatives had not yet matured into a standardized institutional model capable of city-wide scaling. To overcome this limitation, the ARB reframed the policy discourse and catalyzed a new political mobilization network, institutionalizing and expanding the Xinshao experience. In this process, Shaoguan’s functional departments were progressively integrated—differing in depth and scope—into a governance network centered on the Toilet Revolution.

Discourse rescaling laid the cognitive and normative foundation for city-wide mobilization. To elevate the policy’s status, the ARB strategically repositioned the Toilet Revolution from a technical public health intervention to a strategic priority aligned with national development goals. Against the backdrop of the central government’s emphasis on linking poverty alleviation with rural revitalization, the ARB recast the initiative as a vanguard effort in rural development and a safeguard against widespread relapse into poverty. This discursive shift endowed the policy with dual significance—consolidating gains from poverty alleviation and advancing rural revitalization—thereby enhancing its political legitimacy. Crucially, it transformed a sector-specific administrative task into a cross-cutting priority that demanded inter-agency involvement. By reshaping the policy’s meaning, the ARB established a shared cognitive and normative framework across departments, enabling broader coordination. Thus, discourse rescaling served as the foundational mechanism for policy integration in this phase.

The elevation of the inter-agency joint meeting mechanism catalyzed deeper involvement by functional departments. As the political salience of the Toilet Revolution increased, mayors—previously responsible for overseeing implementation at the county and district levels—joined the meetings as key participants, fundamentally reshaping agenda-setting dynamics. By virtue of their institutional authority as leaders of sectoral agencies, their presence transformed the forum from a horizontal coordination platform into a cross-sectoral decision-making body. This shift compelled each department to treat the Toilet Revolution as a core administrative priority. Moreover, several mayors simultaneously held seats on the Shaoguan Municipal Party Committee Standing Committee. Through these pivotal actors, the joint meetings gained direct access to the city’s highest decision-making body, significantly amplifying their political reach. Consequently, the mechanism evolved beyond a deliberative forum into a political mobilization network—one that integrated multiple municipal agencies, bridged top-level party leadership with grassroots implementation, and institutionalized sustained cross-agency alignment around the Toilet Revolution. It was through this network that the Xinshao model was scaled city-wide, transforming localized experimentation into standardized practice across Shaoguan.

The Xinshao case also inspired innovation in procedural policy instruments. Building on this local experiment, the ARB launched the Urban Toilet Open Alliance initiative, showcasing a sophisticated approach to procedural governance. The ARB first collaborated with the Bureau of Justice and the Bureau of Finance to establish an institutional framework that clarified the rights and responsibilities of government and social capital and instituted a risk-sharing mechanism—providing institutional safeguards for private sector participation. To address the shortage and uneven distribution of public toilets in high-density areas such as city centers, tourist attractions, and transportation hubs, the ARB avoided direct administrative intervention, instead using procedural tools to shape outcomes. It set unified standards for sanitation, maintenance, and financial compensation, while granting substantial autonomy to departments such as the HUB and CTB. These agencies were authorized to use government-funded incentives to encourage public and private organizations—including government offices, commercial enterprises, and industrial firms—to open their internal restrooms to the public free of charge. The Urban Toilet Open Alliance exemplifies the strategic use of procedural policy instruments: by establishing a rule-based framework rather than issuing top-down directives, and by integrating funding mechanisms, fiscal incentives, and negotiated coordination, the ARB endowed other subsystems with significant governance resources and discretionary authority. More importantly, it strengthened their recognition of the cross-cutting, boundary-spanning character of the Toilet Revolution. This approach not only enhanced inter-agency cooperation but also significantly expanded the policy’s spatial reach and institutional footprint.

However, this network-embedded model of policy integration—centered on key actors as central nodes—entails profound structural vulnerabilities. Its effectiveness hinges critically on the sustained engagement of these pivotal figures, reflecting a distinctly personalized, even patrimonial, mode of governance. This dependence implies that personnel changes in key positions may rapidly erode the political momentum sustaining network cohesion, thereby exposing the entire actor network to fragmentation or a sharp decline in operational capacity. This network-embedded approach temporarily stabilized integration, though its reliance on key actors rendered it vulnerable to personnel changes—a tension likely to trigger future recalibration.

Although all four modes respond to coordination challenges, they are analytically distinct along two core dimensions: source of authority and degree of formalization. Bureaucratic Pressure-Driven integration (vertical + authority) and Institutional Regulation-Driven integration (horizontal + authority) both rely on hierarchical mandates and codified rules; their difference lies solely in the domain of application—top-down enforcement across administrative levels versus cross-departmental coordination within a single jurisdiction. In contrast, Differential Penetration-Driven integration (vertical + networks) and Network-Embedded integration (horizontal + networks) operate through informal relational channels grounded in trust and social capital. Yet they diverge in directionality: the former propagates centrally endorsed innovations outward through concentric circles of influence, while the latter assembles bottom-up coalitions inward around shared local interests. This analytical distinction clarifies why specific modes emerge under distinct institutional pressures—formal mechanisms tend to dominate when political salience is high and accountability tight, whereas network-based strategies prevail under conditions of resource scarcity, implementation ambiguity, or weak formal coordination capacity.

## 5 Beyond the cognitive framework: comparing governance logics of policy integration in China and the west

### 5.1 A comparative analysis of policy integration types and strategies

The case of the Toilet Revolution in Shaoguan reveals that the essence of policy integration lies in the adjustment of policy relationships. Key subsystems—in this case, the ARB—reconfigure policy frame, policy goals, subsystem involvement, and policy instruments to enhance the overall effectiveness of policy implementation. Specifically, this paper compares four types of policy integration across multiple dimensions, including governance dilemmas, integration strategies, and governance risks (see Table 2).

**Table 2:** A comparative analysis of policy integration types.

Integration type	Bureaucratic pressure-driven integration	Institutional regulation-driven integration	Differential penetration-driven integration	Network-embedded integration
Governance dilemma	Dispersal of policy goals in vertical transmission	Policy silos caused by departmental jurisdictional divisions	Low target identification at the grassroots due to low performance expectations	Difficulty in scaling up successful governance practices
Integration strategy^*^	1. Target responsibility agreements (policy frame) 2. Designated contact system (policy goals) 3. Ritualized practices (policy goals)	1. Formal decomposition of policy goals (policy frame) 2. Agenda-setting mechanism (subsystem involvement)	1. Temporary social funding scheme (policy frame) 2. Leadership endorsement (policy frame) 3. Implementation guidelines (policy instruments)	1. Discourse rescaling (policy frame) 2. Political mobilization network (subsystem involvement) 3. Urban Toilet Open Alliance (policy instruments)
Governance risk	Undermines local adaptive capacity and innovation	Pseudo-integration or innovation suppression due to imbalance among subsystems	Over-reliance on political endorsement	Over-dependence on key actors as central nodes in the mobilization network

* The terms in parentheses refer to the primary dimension of policy integration strategies involved—policy frame, policy goals, subsystem involvement, or policy instruments—following the typology of Candel and Biesbroek (8).

Local governments employ distinct strategies to advance different types of policy integration. While these strategies vary and may enhance governance efficiency in specific contexts, this study does not suggest that one strategy is superior to another. Rather, the strategies discussed here represent rational choices made by local governments in response to specific institutional constraints and policy environments.

### 5.2 Policy frames in comparative contexts: cognitive alignment versus strategic legitimation

In Western governance traditions—particularly the collaborative governance framework articulated by Ansell and Gash (38)—the policy frame refers to how a specific governance issue is perceived. More precisely, when a problem inherently involves multiple functional domains, does the system recognize it as a cross-cutting, complex challenge? And if so, to what extent is there a genuine commitment to pursue an integrated, system-wide approach—rather than allowing sectoral silos to manage it in isolation? Policy frame denotes the dominant and authoritative conception of a governance issue within the highest levels of decision-making and the broader political system. However, a critical misalignment often persists between this centralized understanding and the operational interpretations held by individual sectoral agencies. In the absence of a unifying policy frame to align interpretations and coordinate action, governance systems become vulnerable to systemic failures.

While collaborative governance—as exemplified by Ansell and Gash (38)—represents one influential Western tradition emphasizing cognitive alignment, it is important to note that Western systems themselves vary widely, from federal arrangements with strong subnational autonomy to unitary systems with centralized coordination. Nevertheless, even in centralized Western contexts, legitimacy often derives from participatory consensus rather than top-down authorization. This study reveals a subtle yet significant difference in the functional role of policy frame in policy integration between Western and Chinese governance contexts. While policy frames in typical Western models primarily serve cognitive functions—such as problem recognition and sense-making—those in China’s governance context fulfill a dual role: they not only define problems and establish a shared cognitive foundation for integration, but more importantly, provide a critical source of institutional legitimacy for follow-up efforts.

In Shaoguan case, actor strategies associated with the policy frame—including target responsibility agreements, formal decomposition of policy goals, and discourse rescaling (see Table 2)—played a structurally enabling role in the policy integration process. The former two mechanisms not only defined the responsibilities of subsystems but also endowed them with corresponding authority, thereby clarifying the boundaries of authority and responsibility across agencies and fostering the emergence of a coordinated governance network. The discourse rescaling elevated a single-sector initiative into a citywide political priority, thereby establishing a foundation of institutional legitimacy for subsequent integration. As a result, the policy frame transcended its conventional function of facilitating inter-agency consensus-building, evolving into an institutional infrastructure that provides sustained organizational anchoring and operational logic for policy integration.

As previously discussed, China’s unitary system establishes a highly hierarchical power structure that ensures policy coherence and vertical alignment in implementation. Within this system, consensus formation, elevation, and consolidation around specific governance agendas can often be achieved with considerable efficiency. However, the attainment of such consensus does not automatically translate into the advancement or substantive realization of policy integration. Over time, the *tiao-kuai* structure has entrenched functional segmentation and jurisdictional boundaries between functional departments (“*tiao*”) and local governments (“*kuai*”), with “safeguarding one’s own domain” becoming a de facto norm in everyday administration. When confronted with complex, cross-cutting policy challenges—such as rural public health governance—even in the presence of a clear integrative intent from the higher-level, implementing actors frequently adopt a cautious or even avoidant stance toward inter-agency coordination, driven by concerns over exceeding formal authority or incurring retrospective accountability.

In this context, an institutionalized policy frame serves not only to systematically construct, reinforce, and solidify consensus for policy integration, but more critically, functions as a mechanism of institutional legitimacy. By conferring formal authorization and top-down design, such a framework endows subsystems with procedural legitimacy, thereby mitigating risks of blame and accountability associated with cross-boundary administration. In other words, the institutionalized policy frame operates both as a vehicle for consensus mobilization and as a protective shield against the institutional vulnerabilities generated by the *tiao-kuai* divide.

These findings not only enrich the theoretical understanding of policy integration but also offer the international scholarly community a new perspective on how policy frames operate in non-Western institutional settings. They challenge the conventional Western view that policy frames primarily shape cognition, revealing instead how, in unitary systems, policy frames can actively enable integration through strategic interventions in subsystem involvement, policy goals, and policy instruments. This expands the functional boundaries of the policy frame concept. The implications are significant for building more inclusive and context-sensitive theories of policy integration, particularly in studies of emerging economies or complex governance systems. The Chinese experience provides a critical empirical reference for understanding the diversity and complexity of integration mechanisms in real-world governance. This dual function enables rapid integration during high-pressure periods but also creates dependency on central endorsement; when political attention wanes or leadership changes, the legitimacy shield weakens, precipitating disintegration or strategic re-framing—thus fueling the very fluctuation dynamic this paper seeks to explain.

## 6 Conclusion

This study, through a process-tracing analysis of the Toilet Revolution in Shaoguan, uncovers a central insight long overlooked in the literature: in China’s complex governance architecture, the fluctuations of policy integration may not signal dysfunction, but rather function as an adaptive mechanism. Conventional theories typically frame integration as a linear progression toward stability, coherence, and coordination—centered on the static alignment of goals and instruments. Yet, this paper demonstrates that under conditions of resource scarcity, competing policy objectives, and institutional fragmentation, local governments engage in dynamic adaptation, shifting fluidly between hierarchical pressure and network-based collaboration, between formal regulation and discursive elevation. Far from mere inconsistency, this pattern reflects a strategic balancing act, through which local actors navigate conflicting institutional logics to achieve both political compliance and practical effectiveness.

This insight challenges the conventional understanding of policy integration. In the Chinese context, integration is less about achieving formal uniformity or structural integration than about sustaining policy momentum amid persistent tensions—a form of governance resilience. When rigid top-down evaluations coexist with flexible local realities, standardized mandates with contextual particularities, and departmental boundaries with cross-sectoral demands, fluctuation becomes a source of systemic elasticity.

Theoretically, this paper reframes policy integration fluctuation—not as a deviation to be corrected, but as a constitutive feature of administrative governance under centralized authority. This case suggests that in hierarchical systems with strong central mandates but fragmented implementation capacity—such as China’s—policy effectiveness may not reside in seamless implementation, but in strategic oscillation that enables adaptation without overt defiance. Rather than pursuing idealized models of seamless integration or perfect coordination, future policy design should recognize and institutionalize moderate fluctuation. Creating formal space for local discretion and iterative adjustment can transform apparent instability into adaptive capacity, enhancing the long-term resilience of policy systems.

Together, the four strategies—bureaucratic pressure, institutional regulation, differential penetration, and network embedding—constitute the core repertoire through which local governments generate the adaptive oscillations that characterize policy integration fluctuation.

Admittedly, this study’s findings are grounded in a single in-depth case of the Toilet Revolution in Shaoguan, which limits the generalizability of the fluctuation framework to other policy domains or administrative contexts. Future research can extend this argument in two directions. First, comparative studies across regions and policy domains can examine how institutional contexts shape the repertoire and sequencing of integration strategies. Second, by integrating blame avoidance theory, scholars can probe the micro-foundations of integration fluctuations.

## Footnotes

1 Although many public administration and policy scholars use the concepts of policy integration and coordination interchangeably, such as Tosun and Lang (39), this paper aligns with Candel (40), who views policy integration as an attempt to align policy variables (e.g., goals and instruments), while regarding coordination as alignment at the polity level (i.e., institutional variables).

2 Fiscal Contracting System (*Caizheng Baogangzhi*): A revenue-sharing system (1980s–1993) between central and local governments in China, under which local authorities retained surplus revenues after fixed remittances. While boosting local fiscal incentives, it weakened central fiscal capacity and exacerbated interregional disparities—factors that constrained investment in rural public services, including primary healthcare.

3 Tax Sharing System (*Fenshuizhi*): A 1994 fiscal reform that reclassified taxes into central, local, and shared categories (e.g., VAT as shared), strengthening central revenue. However, by linking local revenues to production-based taxes and introducing tax-rebate transfers, it reinforced local governments’ growth orientation, often at the expense of redistributive priorities such as rural public health provision.

4 The Five Coordinations (*Wuge Tongchou*) encompass: urban–rural integration, regional balance, economic-societal harmony, harmony between humans and nature, and alignment of domestic development with opening-up. Central to this agenda is urban–rural coordination, which prioritizes narrowing disparities in basic public services and rebalancing public resource allocation—particularly in rural education and healthcare. Collectively, these strategies have driven the modernization of China’s rural governance, advancing the equalization and effectiveness of rural public service provision.

5 For the full text of the policy, see “Opinions of the Central Committee of the CPC and the State Council on Implementing the Rural Revitalization Strategy,” February 4, 2018, http://www.gov.cn/zhengce/2018-02/04/content_5263807.htm. Last accessed December 11, 2024.

6 The original policy document is available at https://www.gov.cn/gongbao/content/2019/content_5392301.htm. Last accessed December 3, 2023.
